# Supplementary material for: Structural basis for allosteric regulation of Human Topoisomerase IIα
Source: Nat Commun. 2021 May 20;12:2962. doi: 10.1038/s41467-021-23136-6 (PMC8137924; doi:10.1038/s41467-021-23136-6)
Supplement: Supplementary file 1 — Supplementary Information [file 41467_2021_23136_MOESM1_ESM.pdf]

Supplementary Information

Structural basis for allosteric regulation of Human Topoisomerase IIα

Vanden Broeck *et al.*

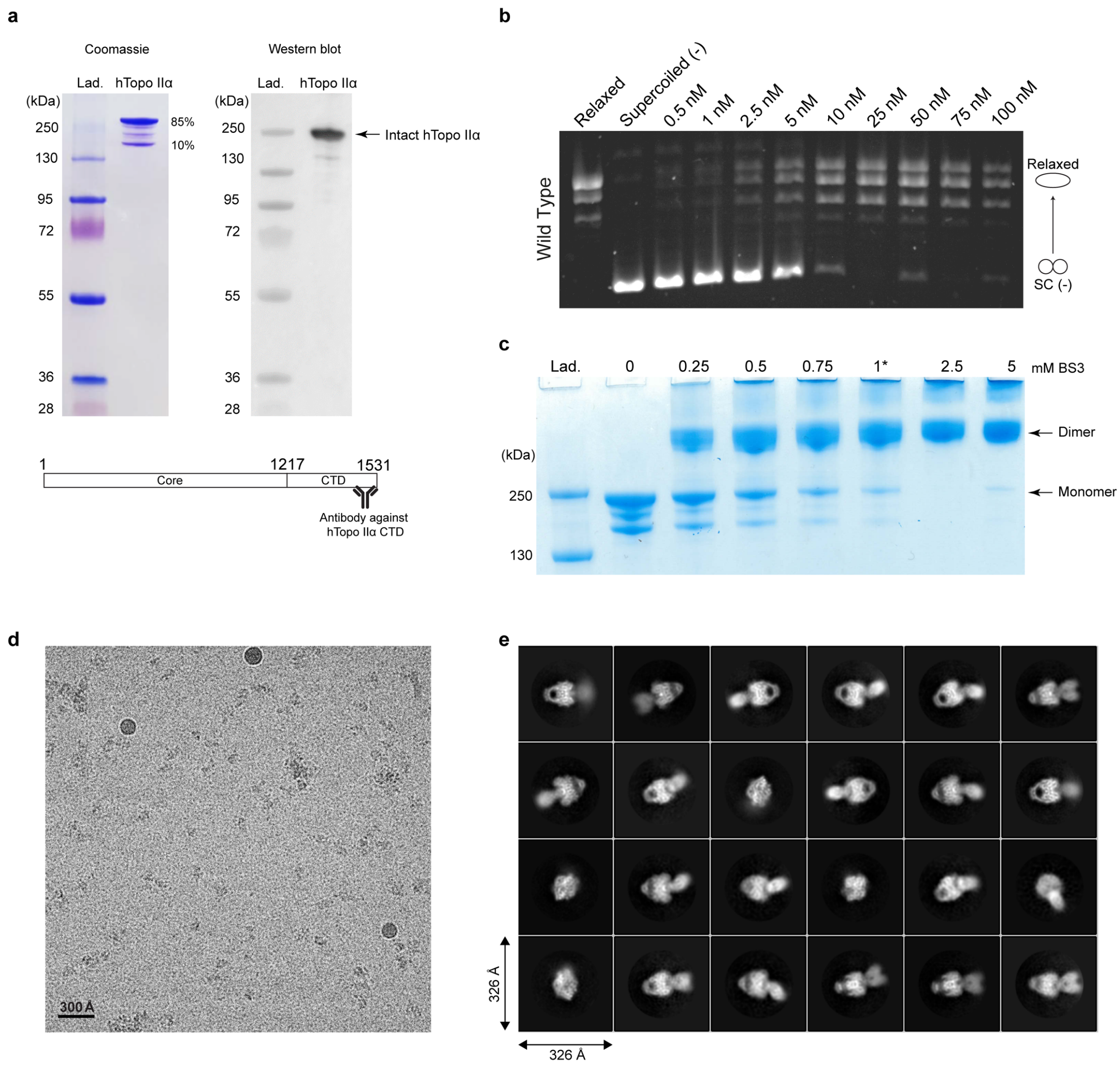

Supplementary Fig. 1. Purification and stabilization of the hTopo IIα. a. SDS-PAGE and Western blot analysis of the purified hTopo IIα. Coomassie staining is shown on the left. Western blot analysis using a monoclonal antibody directed against the hTopo IIα CTD is shown on the right. During the protein preparation, the CTD is cleaved off in 10 to 15% of the sample. Lad. stands for ladder (molecular weight). Three attempts to replicate the purification and western blotting of the hTopo IIα were successful and gave the same protein quality and bands pattern. Uncropped gel and blot are provided in Source Data. **b.** Relaxation activity by wildtype hTopo IIα. Protein concentrations are listed in nM of holoenzyme. Three attempts to replicate the relaxation assay were successful and gave the same bands pattern. Uncropped gel is provided in Source Data **c.** Titration of BS3 for stabilization of the DNA-bound hTopo IIα homodimer. Formation of the full-length complex can still be obtained from the predominant species in presence of DNA and after crosslinking with BS3. The BS3 crosslinking was performed twice and gave very similar results. Uncropped gel is provided in Source Data. **d.** A typical cryo-EM micrograph collected on a Titan Krios microscope operated at 300 kV with a Gatan K2 Summit camera. This micrograph is a representative of the 13,484 micrographs recorded for this study. A total of 6 independent datasets were recorded yielding micrographs with similar overall quality. Scale bar = 30 nm. **e**. Reference-free 2D classification.

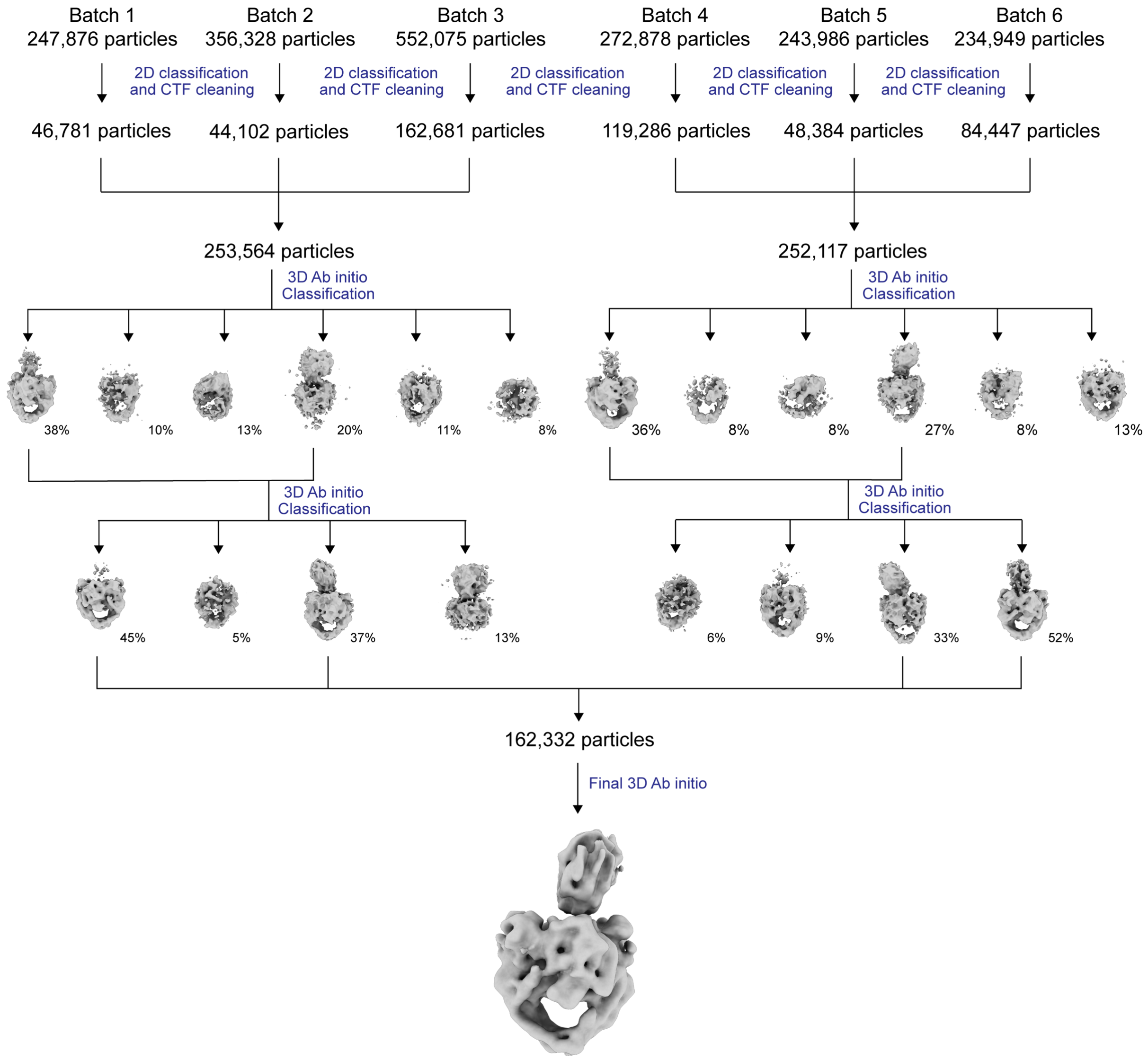

Supplementary Fig. 2. Initial cryo-EM data processing and *ab-initio* model generation. Flow chart of data processing from 2D classification to *ab-initio* model generation in cryoSPARC. The number of particles is indicated at each step.

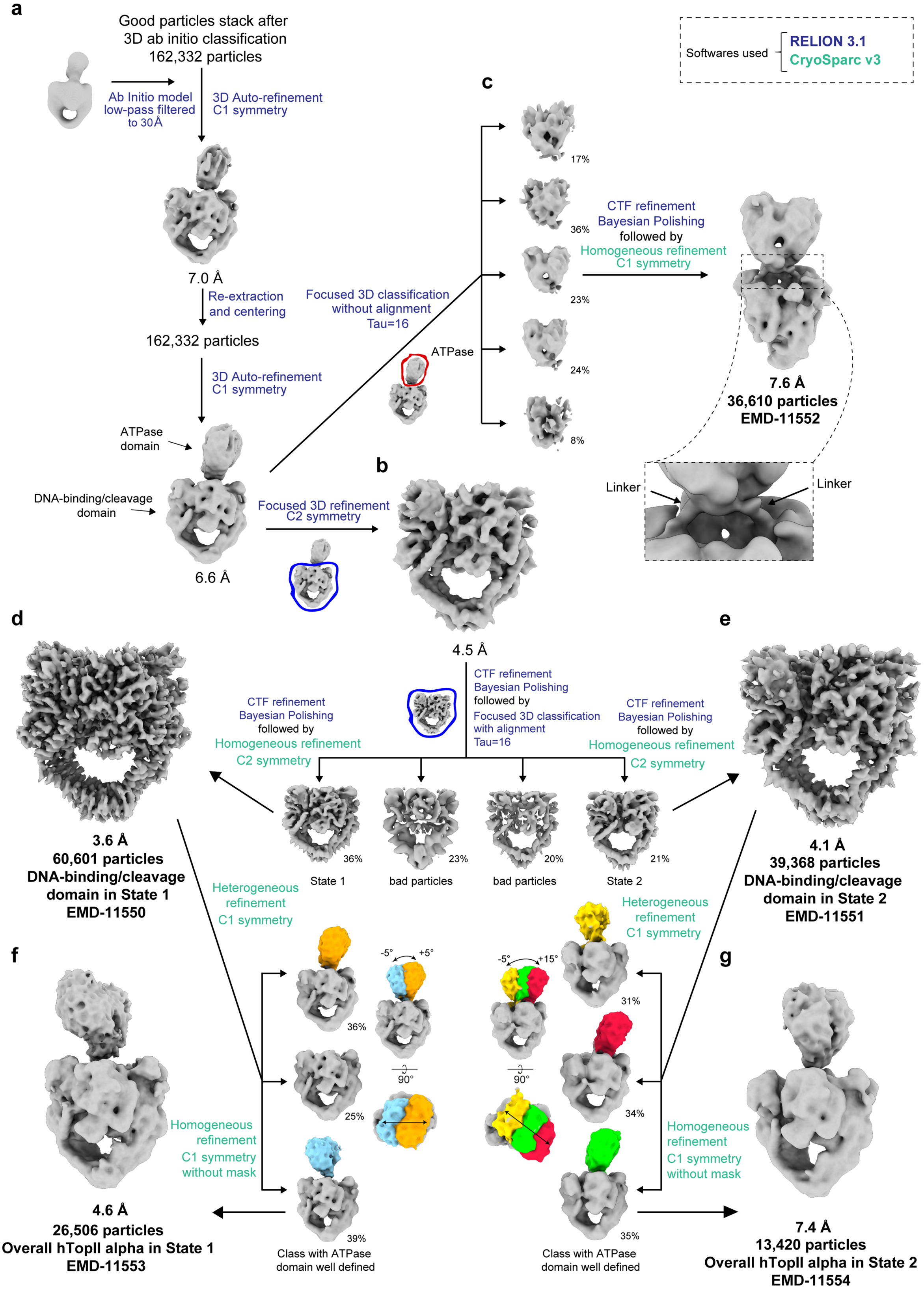

Supplementary Fig. 3. Flow chart of cryo-EM data processing. The *ab-initio* model was refined in cryoSPARC v3 yielding a map of 6.6 Å overall resolution using 162,332 particles (**a**). A subsequent focused refinement on the DNA-binding/cleavage domain yielded a reconstruction at 4.5Å (**b**). Focused 3D classification with and without alignment in Relion 3.1 followed by Homogeneous refinement in cryoSPARC v3 allowed us to solve 5 new

structures: the overall complex with visible linkers at 7.6 Å (**c**) the DNA-binding/cleavage domain in state 1 at 3.6 Å resolution (**d**) and in state 2 at 4.1 Å resolution (**e**), the overall complex in state 1 at 4.6 Å resolution (**f**) (the map is not sharpened to allow better visualization of the ATPase domain) and the overall complex in state 2 at 7.4 Å resolution (**g**). The number of particles used for the final refinement is indicated.

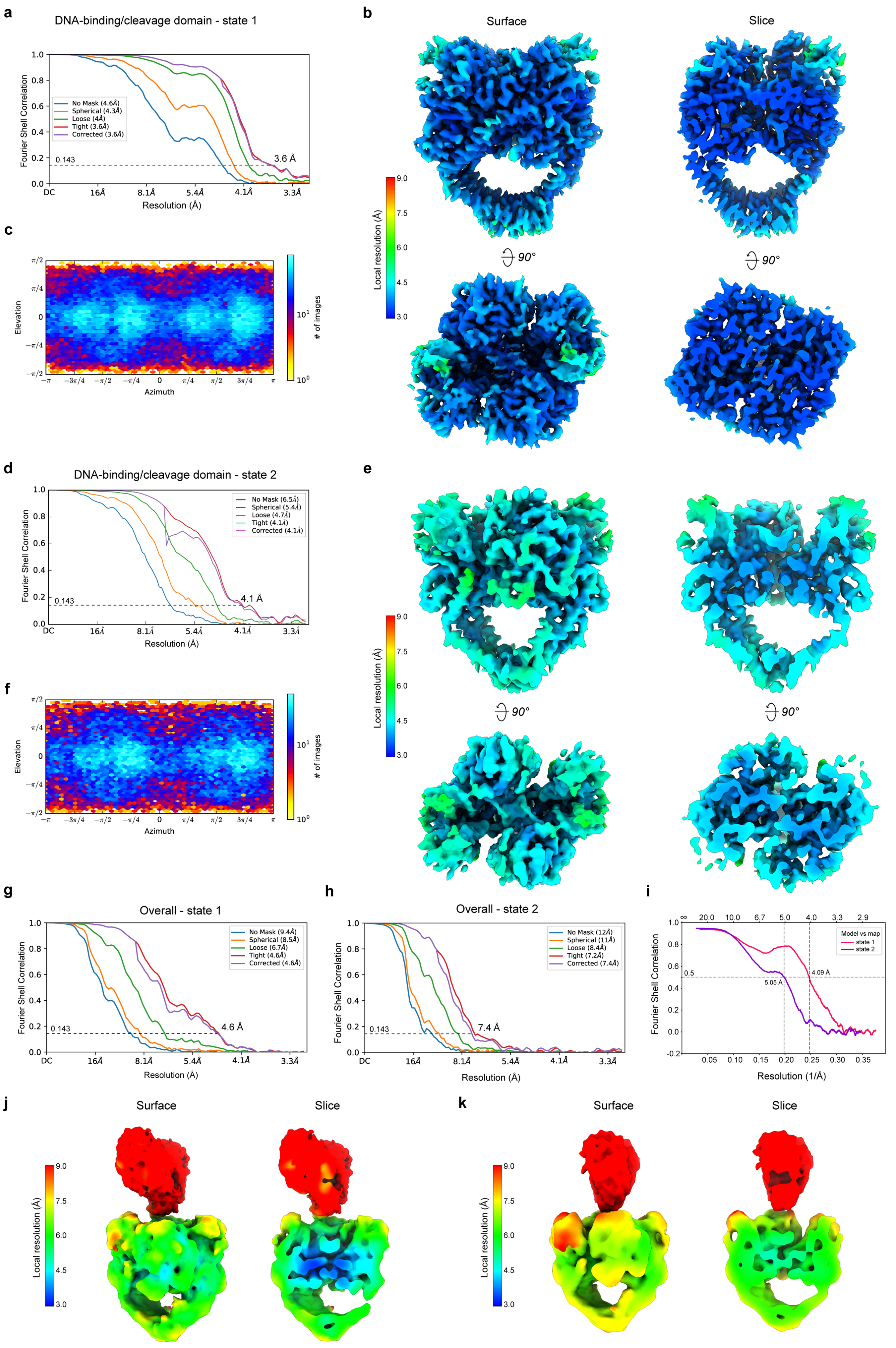
a
DNA-binding/cleavage domain - state 1
Fourier Shell Correlation
Resolution (Å)
No Mask (4.6Å)
Spherical (4.3Å)
Loose (4Å)
Tight (3.6Å)
Corrected (3.6Å)
0.143
3.6 Å
b
Surface
Slice
Local resolution (Å)
90°
c
Elevation
Azimuth
# of images
d
DNA-binding/cleavage domain - state 2
No Mask (6.5Å)
Spherical (5.4Å)
Loose (4.7Å)
Tight (4.1Å)
Corrected (4.1Å)
4.1 Å
e
f
g
Overall - state 1
No Mask (9.4Å)
Spherical (8.5Å)
Loose (6.7Å)
Tight (4.6Å)
Corrected (4.6Å)
4.6 Å
h
Overall - state 2
No Mask (12Å)
Spherical (11Å)
Loose (8.4Å)
Tight (7.2Å)
Corrected (7.4Å)
7.4 Å
i
Model vs map
state 1
state 2
4.09 Å
5.05 Å
Resolution (1/Å)
j
k

Supplementary Fig. 4. Structural data validation and local resolution. FSC plot and resolution estimation using the gold-standard 0.143 criterion (**a**, **d**, **g**, **h**). Particle angular distribution plots (**c**, **f**). Local resolution for the DNA-binding/cleavage domain in state 1 (**b**), in state 2 (**e**) and for the overall complex in state 1 (**j**) and state 2 (**k**) calculated with Blocres. The FSC plots of the atomic models against the cryo-EM maps are shown for the two states for which full models of DNA-binding/cleavage domain were refined (**i**).

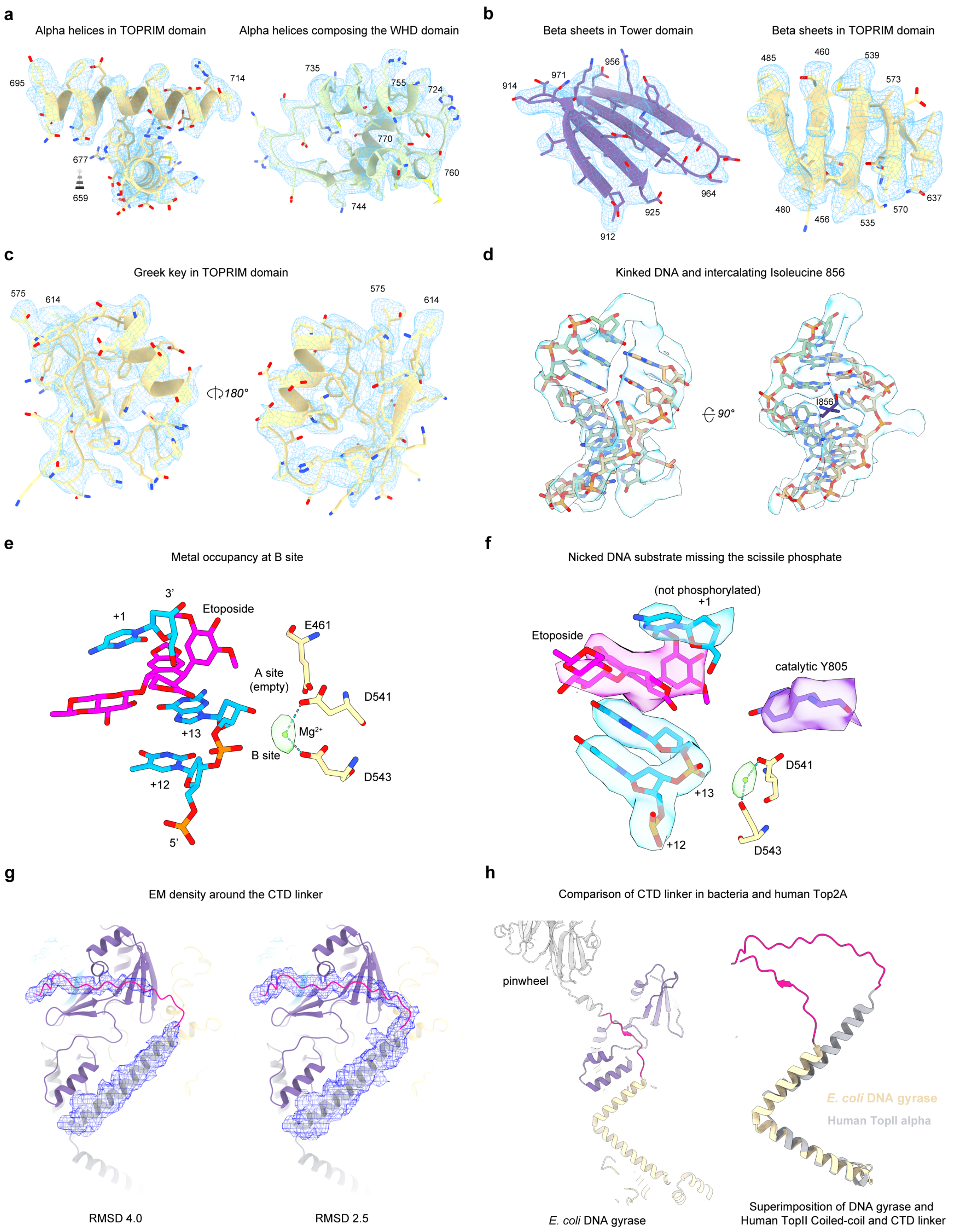

Supplementary Fig. 5. Cryo-EM densities and of the DNA-binding/cleavage domain in closed conformation at 3.6 Å. a. EM density of alpha helices showing well-defined side chains. **b.** EM density showing individual beta strands. **c.** EM density around the Greek key motif in the TOPRIM domain. **d.** EM density around the kinked DNA. **e.** EM density around a magnesium in the B site coordinated by the DxD dyad. **f.** Visualization of the EM density at the catalytic site. **g.** EM density of the CTD linker (magenta line, residues 1191-1215) displayed at two different thresholds **h.** Comparison of the CTD linker path between the bacteria DNA gyrase [1] and human Topo II alpha.

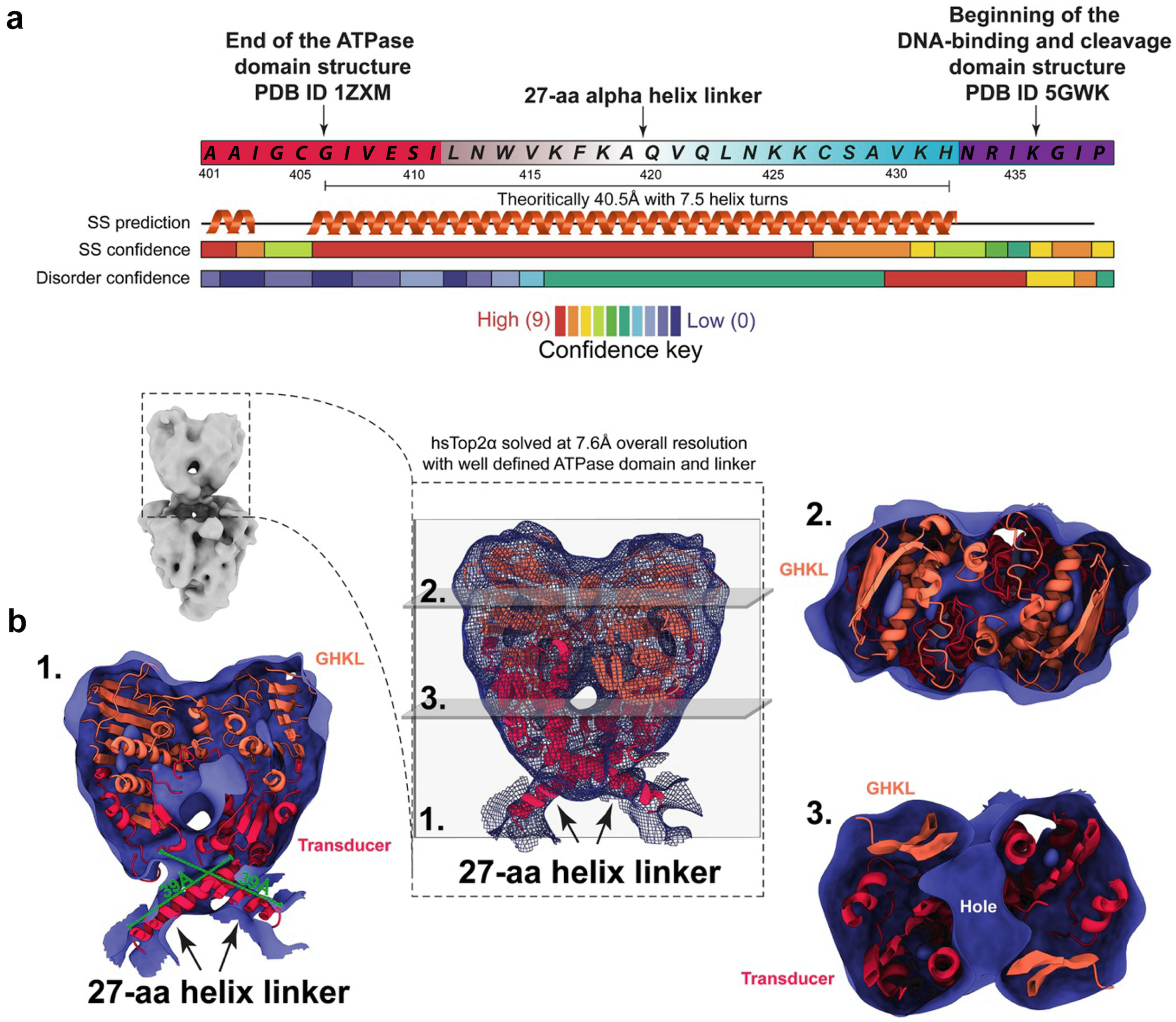

Supplementary Fig. 6. Building of the 27-aa linkers between the N-gate and the DNA gate. a. The 27-aa linkers are predicted to fold as an alpha helix based on secondary structure prediction performed with Phyre2 [2]. The theoretical length of a 27-aa alpha helix is 40.5Å with 7.5 turns, considering that an alpha helix has 3.6 residues per turn and a pitch length of 5.4Å. **b.** After fitting of the functional domains in the EM density, the distance between the last residue of the ATPase domain, C405, (PDB ID 1ZXM[3]) and the first residue of the DNA-binding/cleavage domain, N433, is 39Å. To accommodate such distance with the missing 27-aa, the linkers were built as alpha helices accordingly to the secondary structure prediction and the EM density map. The 3 views of the ATPase domain through different slices of EM density (7.6 Å resolution) show an overall good fit of the atomic model in the map.

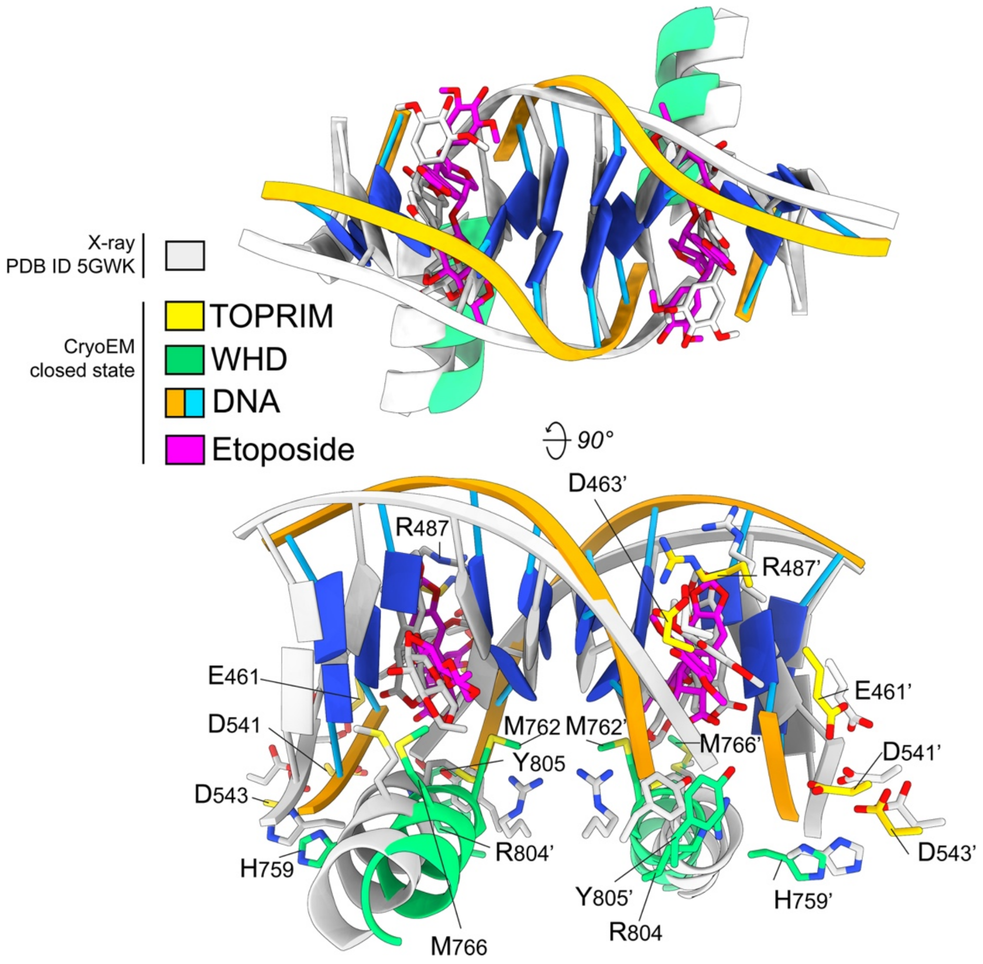

Supplementary Fig. 7. Comparison of etoposide binding sites of cryo-EM and X-ray hTopo IIα structures. Slight rearrangement of the protein structure and DNA bases in the EM structure, compared to the X-ray structure, yielded to a minor shift of the etoposide in the binding site during refinement to avoid steric clashes. Structures are colored as indicated in the legend.

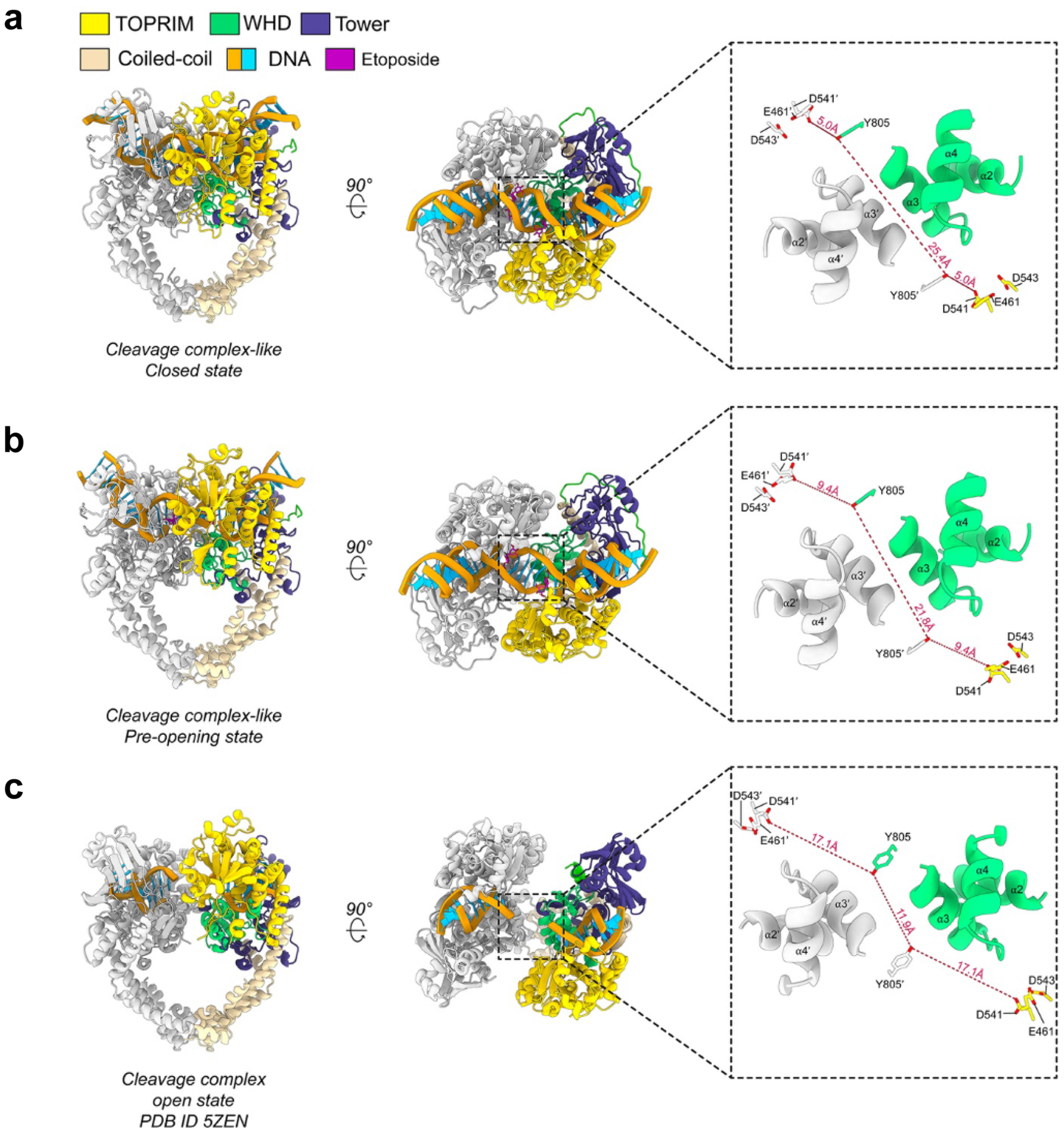

Supplementary Fig. 8. Quaternary and tertiary changes associated with G-segment opening after cleavage. Orthogonal views of the hTopo IIα DNA-binding/cleavage core in different states. The inset shows selected structural elements (alpha helices α2, α3 and α4) lining the bottom side of the G-segment groove and key catalytic amino acids in the closed cleavage complex-like (CC-like) (**a**), pre-opening CC-like (**b**) and open CC (**c**) [4]. For each conformation, the catalytic tyrosine residues, the cation-binding residues and the distances between the two catalytic residues and the closer aspartic acid residue of the DxD dyad are shown to illustrate structural changes in the DNA-gate during closed-to-open transitions. See legend on the top for the color code. Residues from different homodimer are colored differently, with labels belonging to the second homodimer marked by a prime.

Supplemental analysis related to Supplementary Fig. 8

The main conformational changes are observed in the DNA-gate, while the ATPase domain is more prone to rotations and translational movements to accompany the DNA-gate motions (Figure 3). During this conformational transition, the two alpha helices α3 and α3' of each homodimer slide against each other by half helix turn bringing closer the catalytic tyrosine residues rapprochement by 3.6 Å. As the TOPRIM domain performs a swinging outward movement, the DxD di-acidic metal ion-binding motif recede by 4.4 Å from the catalytic tyrosine residues, decoupling the key catalytic residues which disfavor the religation of cleaved DNA ends (Supplementary Fig. 8a-b). Concomitantly to the motion of the DNA-Gate, the dimerized ATPase domain is rotating by 3° counterclockwise (opposite to the intertwining) and is coming closer to the DNA-gate in order to push the T-segment in the newly formed groove between the TOPRIM and tower domains (Figure 3). These two states of the DNA-gate precede the open conformation [3]. In particular, the two alpha helices α3 and α3' of each homodimer have slid against each other by one helix turn, bringing closer the catalytic tyrosine residues. The DxD dyads are now far from the tyrosine residues and follow the sliding and swiveling motion of the DNA-gate (Supplementary Fig. 8c).

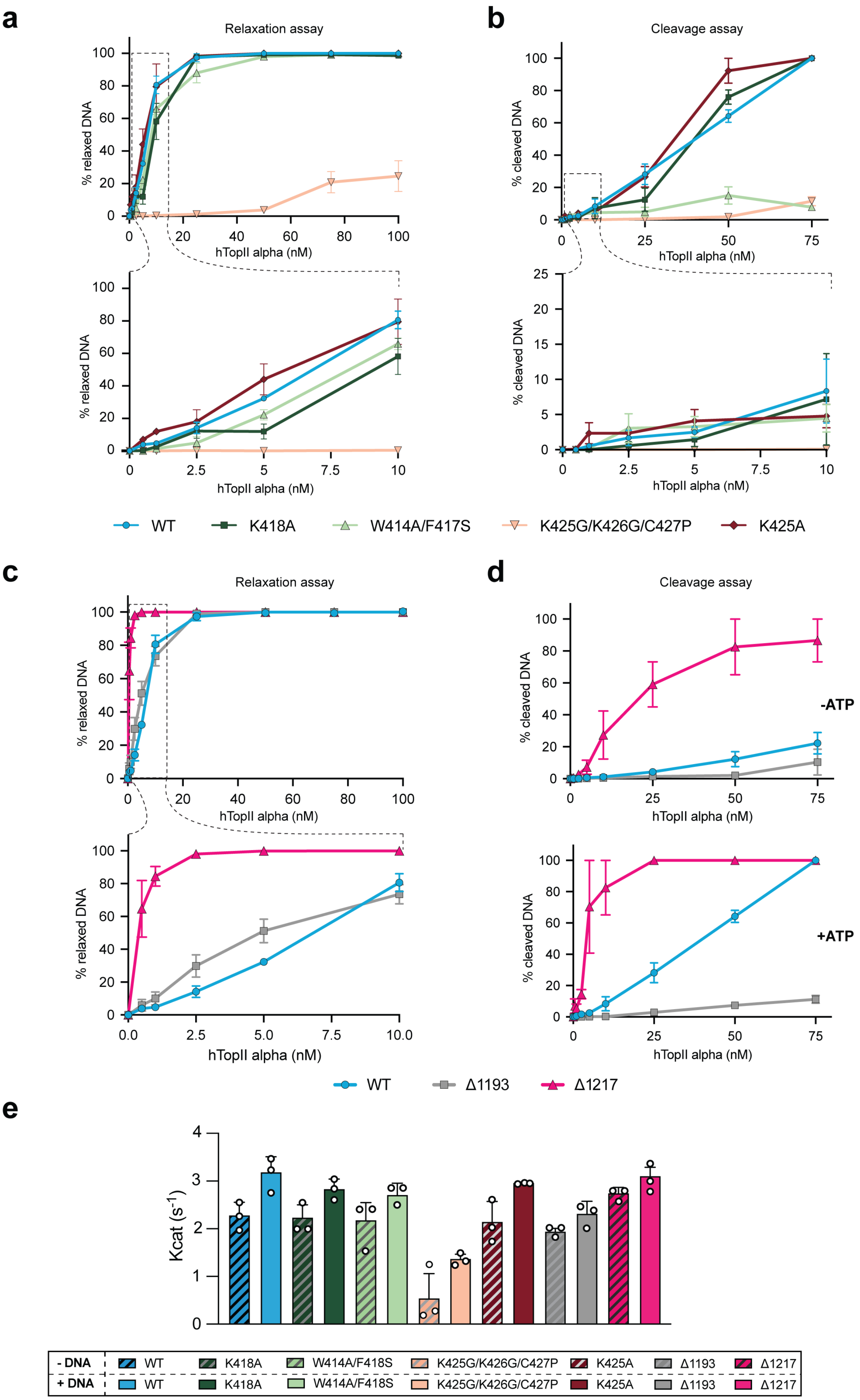
a
Relaxation assay
% relaxed DNA
hTopII alpha (nM)
b
Cleavage assay
% cleaved DNA
hTopII alpha (nM)
WT
K418A
W414A/F417S
K425G/K426G/C427P
K425A
c
Relaxation assay
% relaxed DNA
hTopII alpha (nM)
d
Cleavage assay
% cleaved DNA
-ATP
+ATP
hTopII alpha (nM)
WT
Δ1193
Δ1217
e
Kcat (s-1)
- DNA
+ DNA
WT
K418A
W414A/F418S
K425G/K426G/C427P
K425A
Δ1193
Δ1217

Supplementary Fig. 9. Relaxation, cleavage and ATPase activities of the WT and mutated Human Topo II alpha. a. Relaxation activity of the WT, K418A, K425A, W414A-F417S and K425G-K426G-C427P hTopo IIα. The insert below shows a zoom on the activities between 0 and 10 nM of enzyme. Data are presented as mean values +/- standard error (SE) for 3 independent replicates (n = 3). **b.** Cleavage activity of the WT, K418A, K425A, W414A-F417S and K425G-K426G-C427P hTopo IIα in presence of 275 μM etoposide. The insert below shows a zoom on the activities between 0 and 10 nM of enzyme. Data are presented as mean values +/- SE for 3 independent replicates (n = 3). **c.** Relaxation activity of the WT, Δ1193 and Δ1217 hTopo IIα. The insert below shows a zoom on the activities between 0 and 10 nM of enzyme. Data are presented as mean values +/- SE for 3 independent replicates (n = 3). **d.** Cleavage activity of the WT, Δ1193 and Δ1217 hTopo IIα in absence or presence of ATP. Both assays were performed in presence of 275 μM etoposide. Data are presented as mean values +/- SE for 3 independent replicates (n = 3). **e.** ATPase activities of the WT and mutated hTopo II alpha in absence or presence of DNA. Data are presented as mean values +/- SE for 3 independent replicates (n = 3). Individual data points are also plotted. The source data are provided as a Source Data file.

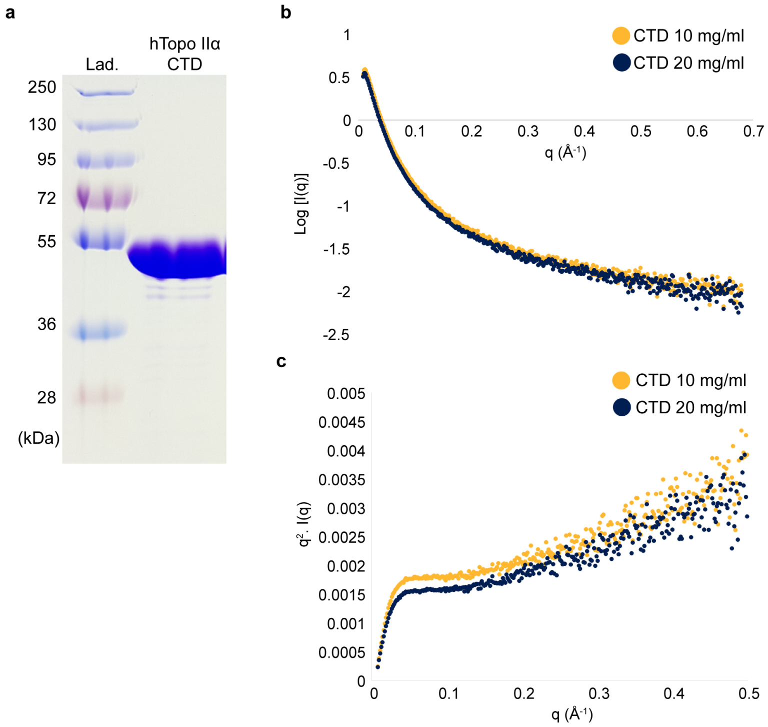

Supplementary Fig. 10. SAXS analysis of the human Topo II α CTD. a. SDS-PAGE analysis of the purified hTopo IIα CTD (1191-1531). Lad. stands for ladder (molecular weight). Uncropped gel is provided in Source Data. Two attempts to replicate the purification of the hTopo IIα CTD were successful and gave the same protein quality and bands pattern. **b.** Experimental SAXS curves of the CTD at 10 mg/ml (yellow) and 20 mg/ml (blue). **c.** Kratky plot demonstrating the absence of fold of the CTD.

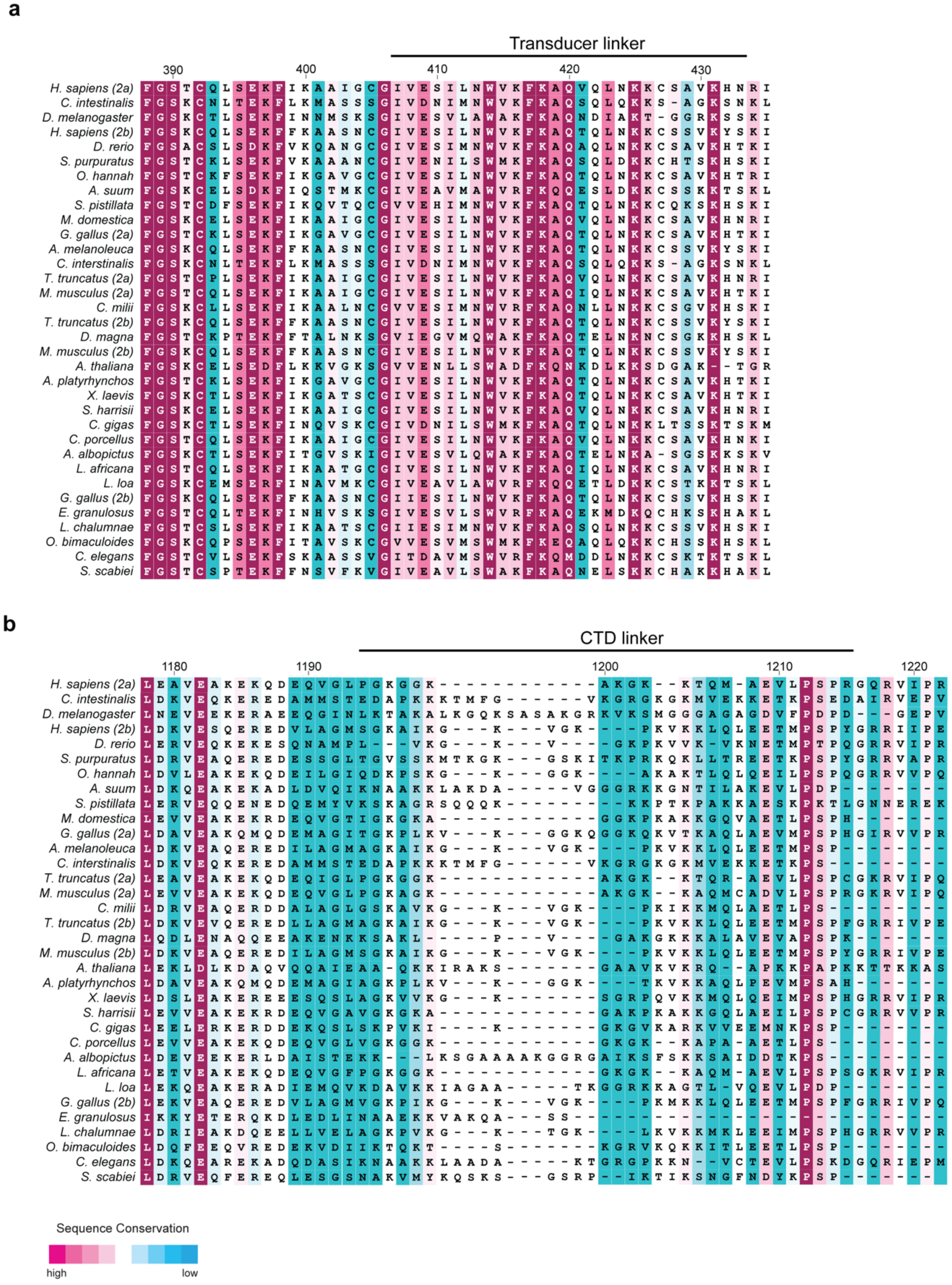

Supplementary Fig. 11. Multiple alignment of metazoans and plant TOP2 proteins. **a.** Focused on the transducer linker. **b.** Focused on the CTD linker. The residues are colored by conservation level: blue is low and red is high.

Data Collection	
Magnification	105,000
Voltage (kV)	300
Electron dose (e^-/Å^{-2})	50
Defocus range (µm)	-1.0 to -3.0
Pixel size (super resolution) (Å)	1.1 (0.55)

Individual data sets						
Batches	**1**	**2**	**3**	**4**	**5**	**6**
Micrographs (no.)	2299	2230	3128	2348	1553	1926
Extracted particles (no.)	247,876	356,328	552,075	272,878	243,986	234,949
Particles after cleaning	46,781	44,102	162,681	119,286	48,384	84,447

Merged data sets for refinement	
Total merged particles	505,681
Particles after cleaning	162,332

Reconstructions				
	**DNA-binding/cleavage domain in state 1**	**DNA-binding/cleavage domain in state 2**	**Overall complex in state 1**	**Overall complex in state 2**
EMDB	11550	11551	11553	11554
PDB	6ZY5	6ZY6	6ZY7	6ZY8
Final particles (no.)	60,601	39,368	26,506	13,420
Pixel size (Å)	1.507	1.507	1.507	1.507
Symmetry imposed	C2	C2	C1	C1
Map resolution (Å)	3.6	4.1	4.7	7.4
FSC threshold	0.143	0.143	0.143	0.143
Map resolution range (Å)	3.1-5.0	3.5-6.0	4.2-10.0	5.5-12.0
Map sharpening B-factor (Å^2)	-110	-171	0	0

Model refinement				
Initial model used (PDB code)	5GWK	5GWK	6ZY5, 1ZXM	6ZY6, 1ZXM
Resolution cut-off (Å)	3.7	4.2	5.0	7.5
Non-hydrogen atoms	13504	13504	19979	19974
Protein/nucleotides residues	1520/60	1520/60	2319/60	2318/60
Ligands	2x EVP	2x EVP	2x EVP, 2x ANP	2x EVP, 2x ANP
Average B-factor (Å^2)	95	210	527	722
R.m.s. deviations				
Bond lengths (Å)	0.011	0.010	0.009	0.009
Bond angles (°)	1.163	1.066	1.020	0.976

Validation				
MolProbity score	1.34	1.30	1.36	1.44
Clashscore (all atoms)	6.20	5.48	6.49	6.95
Poor rotamers (%)	0.15	0	0	0.05
Ramachandran plot				
Favored (%)	98.21	98.41	98.31	97.75
Allowed (%)	1.79	1.59	1.69	2.25
Outliers (%)	0	0	0	0

Supplementary Table 1. Data collection, processing and refinement statistics.

13bp	GAGGATGACGATG
17bp	CGCGCATCGTCATCCTC

Supplementary Table 2. Asymmetric oligonucleotides sequences.

Primer name	Sequence
hTop2a-K418A-fw	AAGCATACTAAACTGGGTGAAGTTTGCGGCCCAAGTCCAG
hTop2a-K418A-rev	CTGGACTTGGGCCGCAAACTTCACCCAGTTTAGTATGCTT
htop2A-W414A-F417S-fw	GTGGTATTGTAGAAAGCATACTAAACGCGGTGAAGAGTAAGGCCCAAGTCCAG
htop2A-W414A-F417S-rev	CTGGACTTGGGCCTTACTCTTCACCGCGTTTAGTATGCTTTCTACAATACCAC
hTop2a-KKC425GGP-fw	GGTGAAGTTTAAGGCCCAAGTCCAGTTAAACGGGGGGCCTTCAGCTGTAAAACATAATAGAATCAAGGGA
hTop2a-KKC425GGP-rev	TCCCTTGATTCTATTATGTTTTACAGCTGAAGGCCCCCCGTTTAACTGGACTTGGGCCTTAAACTTCACC
hTop2a-K425A-fw	AGGCCCAAGTCCAGTTAAACGCGAAGTGTTCAGCTGTAAAAC
hTop2a-K425A-rev	GTTTTACAGCTGAACACTTCGCGTTTAACTGGACTTGGGCCT
hTop2a-Delta1193-fw	GATGAACAAGTCGGACTTGAAGTTCTGTTCCAGGGG
hTop2a-Delta1193-rev	CCCCTGGAACAGAACTTCAAGTCCGACTTGTTCATC
hTop2a-Delta1217-fw	GCCTTCTCCGCGTGGTCTGGAAGTTCTGTTCC
hTop2a-Delta1217-rev	GGAACAGAACTTCCAGACCACGCGGAGAAGGC

Supplementary Table 3. Primer sequences used for the plasmids mutagenesis.

Protein name	Organism	Uniprot ID
TOP2	*Aedes albopictus*	A0A023EXD7
TOP2A	*Ailuropoda melanoleuca*	G1LN78
TOP2A	*Anas platyrhynchos*	U3IFZ2
TOP2	*Arabidopsis thaliana*	P30182
TOP2	*Ascaris suum*	F1KQV5
TOP2	*Caenorhabditis elegans*	Q23670
TOP2	*Callorhinchus milii*	A0A4W3J3D7
TOP2A	*Cavia porcellus*	H0V8L7
TOP2	*Ciona intestinalis*	F7AKG2
TOP2	*Crassostrea gigas*	K1Q404
TOP2A	*Danio rerio*	Q6DRC7
TOP2	*Daphnia magna*	A0A165ADD4
TOP2	*Drosophila melanogaster*	P15348
TOP2	*Echinococcus granulosus*	W6UGB7
TOP2A	*Gallus gallus*	O42130
TOP2B	*Gallus gallus*	O42131
TOP2A	*Homo sapiens*	P11388
TOP2B	*Homo sapiens*	Q02880
TOP2A	*Latimeria chalumnae*	H3ALH9
TOP2	*Loa loa*	A0A1I7VLJ5
TOP2A	*Loxodonta africana*	G3UIA0
TOP2A	*Monodelphis domestica*	F7ABY1
TOP2A	*Mus musculus*	Q01320
TOP2B	*Mus musculus*	Q64511
TOP2	*Octopus bimaculoides*	A0A0L8FFS9
TOP2A	*Ophiophagus hannah*	V8P300
TOP2A	*Sarcophilus harrisii*	G3WFK3
TOP2	*Sarcoptes scabiei*	A0A132ALV4
TOP2	*Strongylocentrotus purpuratus*	W4XMF6
TOP2	*Stylophora pistillata*	A0A2B4S1C7
TOP2A	*Tursiops truncatus*	A0A2U4BNZ1
TOP2B	*Tursiops truncatus*	A0A2U4CFF8
TOP2A	*Xenopus laevis*	Q6INT0

Supplementary Table 4. Uniprot sequence ID of the homologs used for the multiple sequence alignments.

Supplementary References

1. Vanden Broeck, A., Lotz, C., Ortiz, J. & Lamour, V. Cryo-EM structure of the complete E. coli DNA gyrase nucleoprotein complex. *Nat. Commun.* (2019) doi:10.1038/s41467-019-12914-y.

2. Kelley, L. A., Mezulis, S., Yates, C. M., Wass, M. N. & Sternberg, M. J. E. The Phyre2 web portal for protein modeling, prediction and analysis. *Nat. Protoc.* **10**, 845–858 (2015).

3. Wei, H., Ruthenburg, A. J., Bechis, S. K. & Verdine, G. L. Nucleotide-dependent domain movement in the ATPase domain of a human type IIA DNA topoisomerase. *J. Biol. Chem.* (2005) doi:10.1074/jbc.M506520200.

4. Chen, S. F. *et al.* Structural insights into the gating of DNA passage by the topoisomerase II DNA-gate. *Nat. Commun.* (2018) doi:10.1038/s41467-018-05406-y.
