# Supplementary material for: Structural basis for allosteric regulation of Human Topoisomerase IIα
Source: Nat Commun. 2021 May 20;12:2962. doi: 10.1038/s41467-021-23136-6 (PMC8137924; doi:10.1038/s41467-021-23136-6)
Supplement: Supplementary file 3 — Description of Additional Supplementary Files [file 41467_2021_23136_MOESM3_ESM.pdf]

Description of Additional Supplementary Files

File Name: Supplementary Movie 1
Description: Cryo-EM structure of the entire human Topo II α in closed state.

File Name: Supplementary Movie 2
Description: Conformational changes of the human Topo II α from closed to pre-open state.
