# Supplementary material for: Structural basis for allosteric regulation of Human Topoisomerase IIα
Source: Nat Commun. 2021 May 20;12:2962. doi: 10.1038/s41467-021-23136-6 (PMC8137924; doi:10.1038/s41467-021-23136-6)
Supplement: Supplementary file 6 — Reporting Summary [file 41467_2021_23136_MOESM6_ESM.pdf]

nature research

Corresponding author(s): Lamour Valérie

Last updated by author(s): Apr 12, 2021

Reporting Summary

Nature Research wishes to improve the reproducibility of the work that we publish. This form provides structure for consistency and transparency in reporting. For further information on Nature Research policies, see Authors & Referees and the Editorial Policy Checklist .

Statistics

For all statistical analyses, confirm that the following items are present in the figure legend, table legend, main text, or Methods section.

n/a	Confirmed	
☐	☒	The exact sample size (*n*) for each experimental group/condition, given as a discrete number and unit of measurement
☐	☒	A statement on whether measurements were taken from distinct samples or whether the same sample was measured repeatedly
☒	☐	The statistical test(s) used AND whether they are one- or two-sided<br>*Only common tests should be described solely by name; describe more complex techniques in the Methods section.*
☒	☐	A description of all covariates tested
☒	☐	A description of any assumptions or corrections, such as tests of normality and adjustment for multiple comparisons
☐	☒	A full description of the statistical parameters including central tendency (e.g. means) or other basic estimates (e.g. regression coefficient) AND variation (e.g. standard deviation) or associated estimates of uncertainty (e.g. confidence intervals)
☒	☐	For null hypothesis testing, the test statistic (e.g. *F*, *t*, *r*) with confidence intervals, effect sizes, degrees of freedom and *P* value noted<br>*Give P values as exact values whenever suitable.*
☒	☐	For Bayesian analysis, information on the choice of priors and Markov chain Monte Carlo settings
☒	☐	For hierarchical and complex designs, identification of the appropriate level for tests and full reporting of outcomes
☒	☐	Estimates of effect sizes (e.g. Cohen's *d*, Pearson's *r*), indicating how they were calculated

Our web collection on statistics for biologists contains articles on many of the points above.

Software and code

Policy information about availability of computer code

Data collection	The automated date collection program SerialEM (http://bio3d.colorado.edu/SerialEM/) was used for cryo-EM data collection.
Data analysis	All software used for data analysis in this study were available online:<br>1. MotionCor2 (http://msg.ucsf.edu/em/software/motioncor2.html): Image stacks correction;<br>2. Gctf v1.06 (http://www.mrc-lmb.cam.ac.uk/kzhang/Gctf/): CTF estimation;<br>3. RELION 2.1 (http://www2.mrc-lmb.cam.ac.uk/relion): Cryo-EM data analysis<br>4. RELION 3.1 (http://www2.mrc-lmb.cam.ac.uk/relion): Cryo-EM data analysis<br>5. cryoSPARC v0.6.3 (https://cryosparc.com): Cryo-EM data analysis<br>6. cryoSPARC v3 (https://cryosparc.com): Cryo-EM data analysis<br>7. Phenix 1.19 (https://www.phenix-online.org): Model refine<br>8. Coot 0.9 (https://www2.mrc-lmb.cam.ac.uk/ personal/pemsley/coot/): Model building<br>9. ClustalW 2.0 (https://www.ebi.ac.uk/Tools/msa/clustalo/): Multiple sequence alignment<br>10. PSIPRED 4.0 (http://bioinf.cs.ucl.ac.uk/psipred/): Secondary structure prediction<br>11. ConSurf (https://consurf.tau.ac.il): Conservation and structural analysis<br>12. PRIMUS 3.1 (https://www.embl-hamburg.de/biosaxs/primus.html): SAXS data analysis<br>13. UCSF Chimera 1.16 (https://www.cgl.ucsf.edu/chimera/): Density maps and structural models visualization<br>14. UCSF ChimeraX 1.1.1 (https://www.rbvi.ucsf.edu/chimerax/): Structural figures preparation

For manuscripts utilizing custom algorithms or software that are central to the research but not yet described in published literature, software must be made available to editors/reviewers. We strongly encourage code deposition in a community repository (e.g. GitHub). See the Nature Research guidelines for submitting code & software for further information.

Data

Policy information about availability of data

All manuscripts must include a data availability statement. This statement should provide the following information, where applicable:

- Accession codes, unique identifiers, or web links for publicly available datasets
- A list of figures that have associated raw data
- A description of any restrictions on data availability

Model coordinates and density maps from this study are available in the Protein Data Bank (PDB ID 6ZY5, 6ZY6, 6ZY7, 6ZY8) and the EM Data Bank (EMD-11550, EMD-11551, EMD-11552, EMD- 11553, EMD-11554). All other published PDB ID cited in this study are 1ZXM, 5GWK, 4GFH and 5ZEN.

Field-specific reporting

Please select the one below that is the best fit for your research. If you are not sure, read the appropriate sections before making your selection.

[x] Life sciences   [ ] Behavioural & social sciences   [ ] Ecological, evolutionary & environmental sciences

For a reference copy of the document with all sections, see nature.com/documents/nr-reporting-summary-flat.pdf

Life sciences study design

All studies must disclose on these points even when the disclosure is negative.

Sample size	No statistical methods were used to predetermine sample size. As widely accepted in the field, three independent experiments (n=3) were performed for the functional assays and were sufficient.
Data exclusions	Some of the cryo-EM images were excluded after 2D and 3D classifications by following a standard procedure for EM reconstruction. This approach is very common in cryo-EM and allows to discard images that do not contribute to high resolution reconstructions (ice contaminations, denatured particles). No data were excluded for the functional assays.
Replication	For cryo-EM processing, all attempts at replication of the different reconstructions were successful (by using different cryo-EM reconstruction programs that yielded the same EM maps). The replication for each map was performed twice (with RELION 3.1 and cryoSPARC v3). Moreover, the data have been completely reprocessed from scratch during revision and yielded the same maps but at higher resolution. For the functional assays, measurements were recorded three times independently (n=3). The replication was successful within the margin of error. The error bars on the Figures 4c,e,f and 5d,e,f corresponds to the standard error (SE) of the 3 independent measurements. Three attempts to replicate proteins purification and western blotting were successful as they showed identical protein quality and band patterns on SDS-PAGE analysis.
Randomization	The EM data were randomly split into two halves for refinement. Randomization was not relevant for the functional assays. Each individual sample is compared to the others and the sample are not pooled.
Blinding	Blinding was not performed based on the nature of structural biology.

Reporting for specific materials, systems and methods

We require information from authors about some types of materials, experimental systems and methods used in many studies. Here, indicate whether each material, system or method listed is relevant to your study. If you are not sure if a list item applies to your research, read the appropriate section before selecting a response.

Materials & experimental systems

n/a	Involved in the study	
[ ]	[x]	Antibodies
[ ]	[x]	Eukaryotic cell lines
[x]	[ ]	Palaeontology
[x]	[ ]	Animals and other organisms
[x]	[ ]	Human research participants
[x]	[ ]	Clinical data

Methods

n/a	Involved in the study	
[x]	[ ]	ChIP-seq
[x]	[ ]	Flow cytometry
[x]	[ ]	MRI-based neuroimaging

Antibodies

Antibodies used	TOP2A Antibody (1E2 - Sigma-Aldrich) (dilution 1:1000)
Validation	Anti-hTopo IIa mouse monoclonal antibody (1E2) is specific to alpha isoform of human Topo II protein. It was generated using a synthetic peptide directed towards the C-terminal of human hTopo IIa (Sigma-aldrich). The antibody specifically binds to the CTD

of purified hTopo IIa as shown in supplemental fig. 1. The antibody has been successfully used by other labs and cited many times.

Eukaryotic cell lines

Policy information about cell lines

Cell line source(s)	BHK21C13-2P were obtained from the European Collection of Authenticated Cell Cultures (ECACC; 84111301)
Authentication	The cell line was not authenticated.
Mycoplasma contamination	Cells were not tested for mycoplasma contamination.
Commonly misidentified lines (See ICLAC register)	No commonly misidentified cell lines were used in the study.
